# Identifying patients’ signs and symptoms to guide eHealth-directed diuretic therapy intensification in heart failure—Insights from the trial of intensified versus standard medical therapy in elderly patients with congestive heart failure (TIME-CHF)

**DOI:** 10.1007/s00508-025-02551-5

**Published:** 2025-06-17

**Authors:** Arno J. Gingele, Casper Eurlings, Josiane J. Boyne, Hans-Peter Brunner-La Rocca

**Affiliations:** 1 Department of Cardiology, Maastricht University Medical Centre+, P. Debyelaan 25, 6229 HX Maastricht, The Netherlands; 2 Department of Cardiology, Laurentius Ziekenhuis Roermond, Roermond, The Netherlands

**Keywords:** Self-administration, Heart failure, Telemedicine, Signs and symptoms, Diuretics

## Abstract

Heart failure significantly burdens healthcare systems. eHealth products could alleviate this burden by providing patients with recommendations for intensifying diuretic therapy. As evidence on guiding diuretic therapy is scarce, our goal was to identify clinical parameters that eHealth products can use to support heart failure patients in intensifying their diuretic therapy. A post-hoc analysis of the trial of intensified versus standard medical therapy in elderly patients with congestive heart failure (TIME-CHF), comparing an NT-proBNP-guided management strategy with a symptom-guided strategy in elderly heart failure patients, was conducted. Clinical data were collected during patient evaluations at 1, 3, 6, and 12 months of follow-up. At each visit, any increase or new prescription of diuretic therapy was recorded as the outcome event. Mixed-effects logistic regression analyses were used to estimate odds ratios (OR) with 95% confidence intervals (CI), investigating the relationship between the patient data and the odds of diuretic therapy intensification. In this retrospective analysis, 568 heart failure patients were included. Over a 12-month period, 2013 follow-up visits were conducted, during which diuretics were initiated or increased in 370 visits (18.4%). Higher New York Heart Association (NYHA) classification (OR 2.60, 95%CI 1.94-3.50), presence of edema (OR 2.84, 95%CI 2.15-3.77), paroxysmal nocturnal dyspnea (OR 1.52, 95%CI 1.04-2.21), and orthopnea (OR 1.39, 95%CI 1.04-1.85) were significantly associated with the initiation or increase of diuretics. Dyspnea, edema, paroxysmal nocturnal dyspnea, and orthopnea are associated with the intensification of diuretic treatment in HF patients. These symptoms, which patients can easily report, should be integrated into eHealth devices that offer guidance on managing diuretic therapy.

## Introduction

Heart failure (HF) places a substantial burden on healthcare systems [1]. eHealth, which uses information and communication technologies to support healthcare, can empower patients to manage their condition more independently [2]. Although self-care guidelines advise patients to adjust their diuretic therapy during volume overload to prevent deterioration, effective guidance is lacking [3]. Currently, no eHealth solutions are available to support patients in managing this crucial aspect of treatment.

To address this gap, we are developing an eHealth device to support HF patients in self-managing diuretic therapy using parameters that can be collected at home, such as patient-reported signs and symptoms; however, limited evidence for diuretic administration and reliance on healthcare provider judgment in clinical practice make it challenging to establish standardized protocols [4].

To gain information for the development of our device, we conducted a retrospective analysis of ambulatory HF patients to identify signs and symptoms associated with diuretic therapy intensification. These insights should help to integrate effective self-management guidance into eHealth solutions.

## Methods

### Study population

We performed a post hoc analysis of the Trial of Intensified versus standard Medical therapy in Elderly patients with Congestive Heart Failure (TIME-CHF), a randomized controlled trial comparing NT-proBNP-guided versus symptom-guided management in 622 older HF patients (≥ 60 years, NYHA class II or higher) across the ejection fraction spectrum [5]. In the intervention group, NT-proBNP blood levels were targeted to < 400 pg/mL (ages 60–74 years) or < 800 pg/mL (≥ 75 years), while both groups aimed for symptom improvement to NYHA ≤ II. This was achieved primarily through the up-titration of guideline-recommended HF therapy, with additional treatment escalation if necessary. The study adhered to the Declaration of Helsinki, was approved by ethics committees, and included written informed consent.

### Data collection

During follow-up, patients were evaluated at baseline and after 1, 3, 6 and 12 months. Diuretic therapy was initiated or increased upon deterioration, as determined by the treating physician. Signs and symptoms collected at all visits included NYHA class, angina, orthopnea, paroxysmal nocturnal dyspnea (PND), nocturia, edema (self-reported), dizziness, syncope, fatigue, exercise intolerance, cough, constipation, blood pressure and heart rate. The increase or new prescription of diuretic therapy was recorded at each visit as the outcome event. Patients with at least one follow-up visit were included in this analysis.

### Statistical analysis

Descriptive statistics were used to describe the population’s baseline characteristics. To investigate the relationship between collected patient data and the odds of diuretic therapy intensification, mixed-effects logistic regression analyses were performed, estimating odds ratios (OR) with 95% confidence intervals (CI). As each participant could be evaluated multiple times, resulting in repeated observations, a random intercept was estimated for each participant to account for these repeated measures. The regression models were additionally adjusted for age, gender, weight and BNP guidance (yes/no). All statistical analyses were performed using SPSS version 28 (IBM, Armonk, NY, USA).

## Results

### Baseline characteristics

An older population of HF patients (*N* = 568, mean age 76.6 years) with moderate symptoms was included, of whom 40% were female. The most commonly reported comorbidities were hypertension (74%), coronary artery disease (63%), and chronic kidney disease (55%).

### Predictive value of collected items

During the 12-month period, 2013 follow-up visits were conducted. In 370 (18.4%) of these visits, diuretics were initiated or increased. In a multivariable model including all collected items, higher NYHA classification, self-reported edema, orthopnea, higher heart rate, and paroxysmal nocturnal dyspnea (PND) were significantly associated with initiating or increasing diuretics (Table 1). Both diastolic blood pressure and exercise intolerance showed a trend toward association with diuretic therapy intensification but statistical significance was not achieved. No association was found with fatigue or orthostasis.

**Table 1:** Association between patient data and intensification of diuretic therapy

Variable	Odds ratio (95% CI)	*p*-value
Angina	0.94 (0.60–1.46)	0.77
Constipation	0.94 (0.70–1.30)	0.72
Cough	1.09 (0.80–1.48)	0.60
Diastolic BP	1.22 (1.01–1.34)	0.06
Dizziness	0.79 (0.57–1.10)	0.16
Edema	2.84 (2.15–3.77)	< 0.001^*^
Exercise intolerance	1.46 (0.94–2.28)	0.10
Fatigue	0.84 (0.58–1.20)	0.33
Heart rate	1.10 (1.01–1.22)	0.03^*^
Nocturia	1.05 (0.72–1.55)	0.79
NYHA ≥ III	2.60 (1.94–3.50)	< 0.001^*^
Orthopnea	1.39 (1.04–1.85)	0.03^*^
Orthostasis	1.02 (0.76–1.38)	0.88
PND	1.52 (1.04–2.21)	0.03^*^
Syncope	0.52 (0.22–1.27)	0.15
Systolic BP	1.00 (0.90–1.07)	0.74

^*^Significance level: *p* < 0.05

*95% CI* 95% confidential interval, *BP* blood pressure, *NYHA* New York Heart Association functional classification, *PND* paroxysmal nocturnal dyspnea

*OR* odds ratios for heart rate and blood pressure are presented per 10-unit increase

## Discussion

Severity of dyspnea, self-reported edema, orthopnea and PND were significantly associated with intensification of diuretic therapy in a population of older HF patients with moderate symptoms. Conversely, no association was found between fatigue or orthostasis and treatment intensification.

Dyspnea is common in decompensated HF, associated with a poor prognosis and considered a key therapeutic target due to its link with improved outcomes [6]; however, it develops late in deterioration and its absence does not guarantee clinical stability. Similar symptoms, including edema, PND and orthopnea, result from neurohumoral changes, are frequently reported and are predictive for poor outcomes [7]. Additionally, we found that heart rate was associated with diuretic therapy intensification. In HF, decreased cardiac output triggers neurohumoral activation, leading to elevated resting heart rate, which is similarly linked to poor outcomes [8]. Diuretic therapy may alleviate neurohumoral activation by reducing volume overload, which could explain our findings. Thus, HF specialists rely on these typical symptoms and indicators to guide diuretic therapy intensification.

Orthostasis and fatigue, although common in HF and associated with poor outcomes, are primarily related to low blood pressure and impaired cardiac function and may indicate early decompensation [9]. While potentially useful for early detection and intervention, we did not identify them as key predictors of diuretic therapy intensification. Worsening exercise intolerance and elevated diastolic blood pressure were both nonsignificantly associated with diuretic therapy intensification. Reduced cardiopulmonary fitness remains a key marker of poor outcomes, [10] while increased diastolic blood pressure may reflect elevated vascular tone and peripheral resistance in worsening HF. Nonetheless, HF specialists did not place much reliance on these atypical symptoms when guiding diuretic therapy intensification.

Given the limited guidance on diuretic therapy, expert opinion remains the cornerstone for eHealth devices advising HF patients on diuretic treatment. We observed that HF specialists primarily focus on typical symptoms as triggers for diuretic therapy intensification, while placing less emphasis on atypical markers. Although this approach may risk overlooking valuable clinical information reported by patients, prioritizing these typical markers could enhance the practicality of eHealth products designed to guide diuretic therapy. Our next step will involve testing such devices in a pilot study to evaluate their feasibility and clinical utility.

## Limitations

The TIME-CHF study excluded younger HF patients (< 60 years), which limits extrapolation to this group. Furthermore, evaluating the appropriateness of HF specialist decisions to intensify diuretic therapy could have provided clearer insights; however, such data were not collected. Weight changes may be an important trigger for diuretic intensification but were not included in this analysis due to the long and variable time intervals between visits; however, weight as measured during outpatient visits was included as a covariate in our regression model.

## Conclusion

Dyspnea, edema, PND and orthopnea are associated with diuretic treatment intensification in HF patients. These symptoms can be easily reported by patients and may be incorporated into eHealth devices that provide guidance on diuretic therapy.
